# Optimization of Buckypaper-enhanced Multifunctional Thermoplastic Composites

**DOI:** 10.1038/srep42423

**Published:** 2017-02-13

**Authors:** Zhongrui Li, Zhiyong Liang

**Affiliations:** 1 Electron Microbeam Analysis Laboratory (EMAL), University of Michigan, MI 48109, USA; 2 High Performance Material Institute, Florida State University, Tallahassee, FL 32310, USA.

## Abstract

A series of flattened-nanotube reinforced thermoplastic composites are sizably fabricated as a function of buckypaper loading. The effects of the volume fraction, nanotube alignment and length on the tensile performance of the composites are factored into a general expression. The incorporation of self-reinforcing polyphenylene resin (Parmax) into a highly aligned buckypaper frame at an optimal weight ratio boosts the tensile strength and Young’s modulus of the buckypaper/Parmax composite to 1145 MPa and 150 GPa, respectively, far exceeding those of Parmax and aligned buckypaper individually. The composite also exhibits improved thermal (>65 W/m-K) and electrical (~700 S/cm) conductivities, as well as high thermoelectric power (22 μV/K) at room temperature. Meanwhile, the composite displays a heterogeneously complex structure. The hexyl groups of Parmax noncovalently interact with the honeycomb structure of the flattened nanotube through π-stacking and CH-π interaction, correspondingly improving the dispersity of polymer on the nanotube surface and the interfacial stress transferring while the high alignment degrees of nanotube facilitate phonon and charge transport in the composites.

The quest for structural materials that are multifunctional and lightweight is important for future technological and engineering advances. The unique combination of remarkable axial mechanical, electrical and thermal properties makes carbon nanotube (CNT) among the most promising materials for the renovation of a wide range of applications. For instance, an ideal single wall CNT has half the mass density of aluminum, 20 times the tensile strength of iron, a 10-fold increase over the electron mobility of silicon, and can carry 1000 times the maximum current density of copper wire. CNT also exhibits 5 times the thermal conductivity of Cu. Such outstanding properties make it a clear front-runner for use in future technologies. Sharing many similar morphological features of polymers such as high aspect ratio and high surface area, CNT has been extensively used as nanofiller in polymer matrix in an attempt to fabricate multifunctional composites^1^.

The superior mechanical properties of individual CNT alone, however, do not ensure the composite’s superior strength, stiffness, and fracture toughness. Composites using un-oriented CNTs dispersed in polymers exhibit only marginal tensile property improvements at low CNT content^2^. The homogeneous dispersion of CNTs throughout the matrix without destroying the integrity of the CNTs is crucial to the effective utilization of nanotubes in composite applications. In practice, the development of CNT composites has been hindered by difficulties (such as the aggregation of nanotubes at higher concentrations and phase segregation between nanotubes and polymers) in dispersing CNTs in polymers at high weight fractions (at least 20–60 wt% or more CNTs) while achieving uniform and strong interactions with the polymer matrix^3^. CNTs must take part in the load transfer through good interfacial interaction. Performance of CNT composites also critically depends on the effectiveness of the interfacial stress transfer, which, in turn, depends on the nature and strength of the nanotube/matrix interface. The misalignment and agglomeration of the CNTs in the buckypaper can weaken their mechanical strength^4^ and thermal conductivity^5^. High resistance at nanotube contacts significantly undermines the electrical conductivity of the buckypaper.

Currently available analysis of composite is based on a single volume fraction^6^, which was verified experimentally by very few studies on the mechanical properties of polymer-nanotube composite at relatively low ranges of nanotube contents^7^,^8^. However, very little details are known about the optimal CNT loading for different physical properties of the final composites. Without the knowledge of the optimal CNT content level, it is impossible to determine the maximum achievable modulus and strength of the hybrid composites. Furthermore, tube orientation distribution and statistical tube length are often simply approximated to proportional to a Krenchel’s orientation efficiency factor, η_o_^9^, and a length factor, η_l_^10^. However the Cox–Krenchal rule of mixtures has not been fully validated for any composites with a wide range of orientations or long tubes.

Besides the CNT content, length and orientation factors, the micro/nano structure of composites should also plays important role in their performance. Nature offers a great structure source for templates of multifunctionality. Many biological materials such as shells of abalones, cortical bone and nacre, are ingeniously produced as lightweight, strong, and high-performance materials with many exceptional functionalities^11^. This is exemplified by nacre (mother of pearl), which consists of high inorganic content (almost 95 vol % calcium carbonate) and low elastic biopolymer proteins. The platelet-shaped aragonite crystals and proteins are layered into a “brick-and-mortar” structure, which is the key to nacre’s outstanding mechanical properties.

In this work, we developed a solution-hot-press approach to sizably fabricate high performance buckypaper-reinforced composites by mimicking the “brick-and-mortar” structure using highly aligned CNTs (“bricks”) and self-reinforcing polyphenylene resin (Parmax, “mortar”), see Fig. 1. For engineering applications such as macroscopic structural applications with high mechanical and electrical performances, CNT-reinforced polymer composites must be large enough to be commercially applicable, and the manufacturing approach must be affordable and scalable in terms of production capability and product size. As a 2D network assembly of CNTs, buckypaper can be fabricated in a very large size. The intrinsic properties of buckypaper carried on from individual CNTs make them very useful in broad fields such as catalyst supports, actuators, battery electrodes, capacitors, filtration, and thermal and electrical conductors. As a liquid crystalline polymer, Parmax is a copolymer of para-linked benzophenone and meta-linked unsubstituted phenylene units^12^. As a self-reinforced polymer with a large molecular weight (26,900–30,000), Parmax has high mechanical strength (~207 MPa), tensile modulus (5.5 GPa) and rockwell hardness (80 B), as well as outstanding thermal stability^13^. The solution impregnation and hot-press process enables a homogeneous dispersion of polymer chains on the buckypaper. The strong affinity of Parmax to buckypaper enhances the interfacial binding between Parmax matrix and CNTs. The tensile properties, thermal conductivity and electrical conductance of the composites were systematically investigated as a function of CNT loading.

## Materials and Methods

### Materials

The buckypaper sheets (purchased from Nanocomp Technologies Inc., Concord, New Hampshire USA) contain the multi-walled carbon nanotubes (MWNTs) with lengths of several hundred micrometers. To align the CNTs in the buckypaper, a resin (Hexcel 8552) was pressed into the CNT sheet (size 30 cm × 45 cm) using the hot-press at 20 tons and 80 °C for 1 hour, and the strips were subject to a consistent and slow uniaxial strain under ~65 °C heating until 65% elongation was achieved (*i.e*., the post-stretched sheet is 65% longer than the pre-stretched one). The removal of the resin was performed by immerging the prepreg in acetone for 2 days and followed by washing with dilute acid and water^14^. Only a trace amount of the resin (<0.5 wt%) was found in the purified buckypaper strips. The liquid crystalline polymer used in this work was poly [(benzoyl-1, 4-phenylene)*-co-*(1, 3-phenylene)] trademarked as Parmax^®^, obtained from Mississippi Polymer Technologies Inc.

### Composite Preparation

Parmax pellets were first dissolved in dimethylformamide (DMF) with the assistance of sonication. To make buckypaper/Parmax prepregs of different CNT loading, different amounts of the Parmax/DMF (0.5 mg/ml) solution were dipped onto the stretched buckypaper, and dried at 80 °C in a vacuum oven for 12 hours to remove the adsorbed DMF solvent. Then the buckypaper prepregs were subjected to a pressure of approximately 2.0 MPa at 290 °C for 30 minutes, and after then allowed to cool to room temperature. The weight fractions of CNTs in each composite sample were calculated using the weight of the buckypaper divided by the total mass of the final composite samples. The final samples were named as *xBPmx (x* is the weight percentage of buckypaper in the composite).

### Characterization

The surface and cross sectional morphologies of the composite samples were examined using a field emission scanning electron microscope (JEOL 7401 F) with a beam voltage of 10 kV. The small and wide angle X-ray scattering (SAXS/WAXS) measurements were performed on a Bruker NanoSTAR system with an Incoatec IlS microfocus X-ray source operating at 45 kV and 650 μA. The primary beam was collimated with cross-coupled Gobel mirrors and a pinhole of 0.1 mm in diameter, providing a Cu *Kα* radiation beam (λ = 0.154 nm) with a beam size about 0.15 mm in full width half maximum (FWHM) at the sample position. The small-angle scattering intensity was measured on a two-dimensional multiwire Hi-STAR detector. The wide-angle diffraction intensity was captured by a Fuji Photo Film image plate, and read with a Fuji FLA-7000 scanner. The Fourier transform infrared spectroscopy (FTIR) data of the samples were obtained in transmission mode on Nicolet Magna IR-860 FTIR spectrometer. The specimens were mixed with KBr powder and pressed into a 1-cm disc (~0.5 wt% CBT) and placed on the sample holder. A background absorption spectrum was taken before each run and subtracted from the sample spectrum. All spectra were recorded from 400–4000 cm^−1^. A total of 32 scans at a resolution of 2 cm^−1^ were averaged.

The composite strips for tensile tests were cut with dimensions of approximately 50 mm (L) × 5 mm (W) × 20~50 μm (D). Tensile performance was evaluated using a Shimatsu AGS-J materials testing system (Kyoto, Japan) at room temperature (23 ± 2 °C) and 40 ± 5% relative humidity, with a crosshead speed of 1 mm/min on a 500 N load cell. At least five specimens of each composite type were tested, and the results were averaged to ensure reproducibility. Considering the cross-sectional area decreasing and necking, true stress/strain curves are obtained by the instantaneous load acting on the actual cross-sectional area and assuming material volume remains constant^15^. The interfacial interactions of tube-polymer (Y_tp_), tube-tube (Y_tt_) and polymer-polymer (Y_pp_) in the buckypaper/Parmax composites were estimated by fitting the Young’s module *vs* interpolating factor curve using the equation set 3 along with the measured interpolating factor, tube length and orientation factors for the composites. The temperature dependent mechanics of the composites was studied using dynamic mechanical analysis (DMA) on a TA Instruments DMA Q-800 working at a fixed frequency of 1 Hz in the tensile mode. Rectangular shaped samples of 20 mm long and 4 mm wide were mounted in a large tension clamp. For each sample, the temperature was ramped from 50 °C to 300 °C, at a 3 °C/min heating rate. The in-plane electrical and thermal conductivities of the stretched-buckypaper/Parmax composites were measured along the stretching direction using a physical property measurement system (PPMS, Quantum Design). The samples, typically ~0.05 mm thick, were cut into 10 mm (stretching direction) × 2 mm strips, and the probe distance was about 5 mm. A resistive heater and a temperature sensor were attached to one end of a sample through a metal lead using thermally conductive silver epoxy, while the other end was attached to a cold foot and a second temperature sensor. At a given power setting of the heater, the temperature difference between the two sensors was used to calculate the in-plane thermal conductance, and the thermal conductivity was calculated by the dimension. High vacuum and radiation shields were used to minimize the heat loss from the heater. The electrical conductivity was measured afterward with the same two-probe contacts at the same temperature.

## Results and Discussion

### Multifunctionalities

#### Electrical and Thermal Conductivities of buckypaper/Parmax Composites

**Figure 2:**
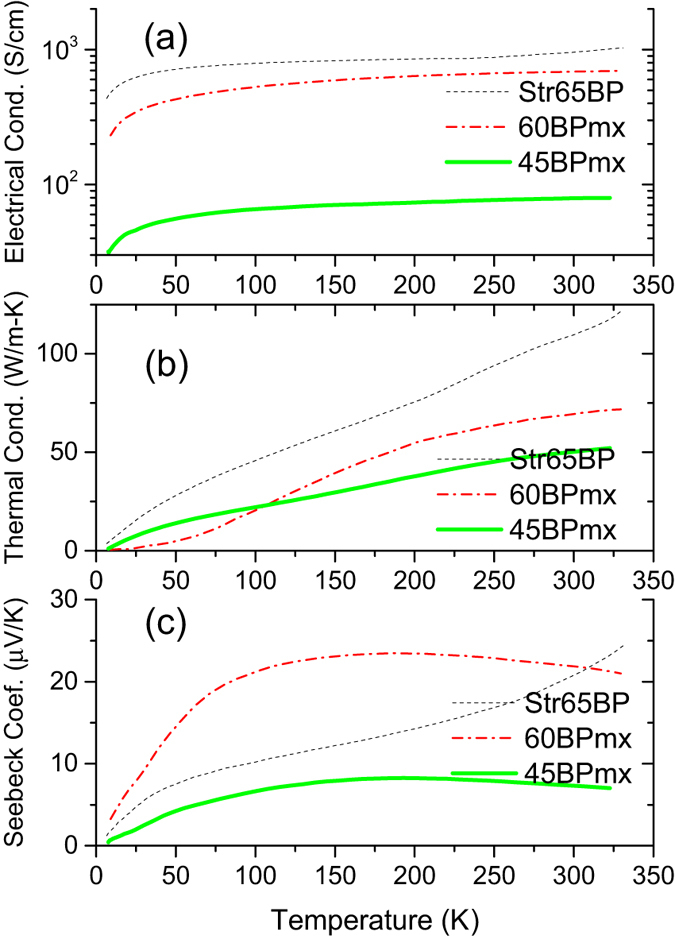
The temperature dependence of thermal conductivity (**a**), electrical conductivity (**b**) and Seebeck Coefficients (**c**) of the buckypaper/Parmax composites with different carbon nanotube content. The thermal and electric properties of the 65%-stretched buckypaper are also included for comparison.

CNTs are excellent filler for the fabrication of conductive nanocomposites thanks to their high charge mobility and high aspect ratio. The dispersion and alignment of MWNTs in the polymer matrix directly determine the electrical properties of the polymer composites^16^. As seen in Fig. 2a, the electric conductivity (σ) of the buckypaper/Parmax composites along with 65%-stretched buckypaper strip shows a positive temperature dependence (dσ/dT > 0), showing a non-metallic behavior. The mechanisms of the charge carrier transport in the buckypaper network are mainly the fluctuation-assisted tunneling through barriers and the variable-range hopping between mesoscopic metallic islands of conducting tubes separated by insulating ones^17^. Parmax is an insulator with an electrical conductivity of approximately 10^−13^ S/cm. The electrical conductivity is dramatically enhanced by the incorporation of buckypaper. When 45 wt% CNT is added to the Parmax (*45BPmx*), the conductivity increases to 78.7 S/cm, improved approximately 14 orders of magnitude compared to that of the neat Parmax. For the composite *60BPmx*, the conductivity climbs to 698.5 S/cm, significantly higher than that of the composites containing low CNT content by using the regular mixing dispersion approach^18^. These high conductivities are attributed to the MWNT alignment and the dense packing of MWNT buckypaper, leading to better contacts among the nanotubes. The electric conductivity of the neat 65%-stretched buckypaper sample can reach 1032.3 S/cm.

CNTs are also well-known for their excellent phonon transport capabilities with an experimentally measured individual-nanotube thermal conductivity (*k*) of above 3000 W/m-K^19^, which have led to excitement about their potential use in polymeric composites with high thermal conductivities^20^,^21^. As seen from Fig. 2b, from 5 to 325 K, the thermal conductivity of the 65%-stretched MWNT buckypaper (*Str60BP*) increases smoothly as the temperature increases, and displays a temperature dependence similar to those of random^22^ and aligned SWNT buckypaper^23^. They typically increase parabolically at the low temperature range, linearly at medium temperature range and show an upturn of thermal conductivity at room temperature^24^. At room temperature, the thermal conductivity of the 65%-stretched MWNT buckypaper strip exceeds 100 W/m-K, comparable to that of diamond or graphite^25^. The higher *k* values of better-aligned nanotubes than graphite might be due to the low dimensionality of nanotubes which might suppress the Umklapp processes^26^. Interestingly the buckypaper/Parmax composites show a saturation of thermal conductivity near room temperatures (>250 K) due to the reduced radiation from the surface and onset of the Umklapp process. As expected, the thermal conductivity of the composite decreases as the less CNTs in the composites, since Parmax is a poor thermal conductor, and phonon modes within CNTs can be damped and scattered by the more polymer which reduces the thermal conductivity of the CNTs^27^. Similar observations were also reported for the thermal conductivity of the graphite composites, which drops almost linearly with the decreasing graphite content^28^. Compared with other thermoplastic composites such as PEEK and PPS based composites (1.8~2.8 S/cm and 1~4 W/m-K) of the similar buckypaper content^29^, our composites have much higher electric conductivity as well as thermal conductivity because of the longer and highly aligned CNTs in our composites.

The electron contribution to the thermal conductivity can be determined by measuring the thermal and electrical conductivities of a material. The Lorenz ratio *k*/σT of the 65%-stretched buckypaper strip, has a value of 7 × 10^−6^ (V/K)^2^ at 300 K, which is more than two orders of magnitude greater than the value expected for electrons and consistent with the phonon-dominated thermal conductivity of CNTs over the measurement temperature range. Furthermore, for the buckypaper/Parmax composites, the Lorenz ratio declines with increase in the CNT content, suggesting that the electrical conductivity is likely more affected by the CNT content in the composites than the thermal conductivity.

Owing to its nanoscale, low-dimensional, and holey structural features^30^, individual MWNT (Seebeck coefficient S = 80 μV/K at 300 K) and SWNT (S = 40 μV/K at 300 K) also exhibit excellent thermoelectric properties with proper metal contact (If electrons in a nanotube transport ballistically, then the Seebeck coefficient of the nanotube should be zero). The thermoelectric power (TEP) values of the neat buckypaper strip and the buckypaper/Parmax composites are positive and increase with temperature (Fig. 2c), a typical characteristic of a moderately *p*-doped semiconductor in which the holes dominate the carrier transport. The *p*-type characteristics might indicate the formation of new localized acceptor states in the valence band^31^. Like the annealed SWNT^32^, the neat 65%-stretched buckypaper exhibits an escalated TEP, even above room temperature, which differs from the linear temperature dependence reported for an individual nanotube^33^. This nonlinearity might stem from Kondo effects induced by magnetic catalytic particles in the buckypaper^34^, the parallel transport through semiconducting tubes^35^, and/or the phonon drag effect (additional charge carriers dragged from the hot to the cold end by phonon flux via momentum transfer)^36^. The Seebeck coefficient value (S ≈ 25 μV/K) of the 65%-stretched buckypaper strip at room temperature is lower, which can be attributed to the orientation of inter-tube barriers relative to ΔT^37^. This result is consistent with the other’s observation that randomly oriented nanotube network has a larger S compared to aligned CNTs^38^.

The ability of a given material to efficiently produce thermoelectric power is related to its dimensionless figure of merit (ZT = S^2^σT/κ), which is linearly proportional to the square of the Seebeck coefficient (S), the operating temperature (T), and the electronic conductivity (σ), and inversely proportional to the thermal conductivity (κ). The thermal conductivity can be reduced and the relationship between σ and κ can be slightly decoupled by introducing the electrically insulating Parmax molecular chain into the tube-tube junctions. Because of the low thermal conductivity and amorphous, randomly-oriented structure, a thin layer of Parmax coating on CNT can serve as a phonon scattering point while still allowing the electronic charge carriers to hop across the barrier. Clearly, incorporating proper amount of Parmax polymer can boost the thermoelectric performance. For example, the Seebeck coefficient of *60BPm* is much higher than that of neat buckypaper below 300 K. Furthermore, the high Parmax loading would result in the decrease of the Seebeck coefficient of the buckypaper/Parmax composite. For instance, the Seebeck coefficient value (S = 7.2 μV/K) of the composite *45BPmx* (55 wt% Parmax) is only 32% of that of *60BPmx* (40 wt% Parmax). With the increase in Parmax content, the Parmax coating layer becomes thicker, causing the quantum states in the quantum region normal to the axis of the nanotube to decrease, which would weaken the energy-filtering effect. Additionally, the emerging Parmax clusters also weaken the Seebeck coefficient enhancement, indicating the presence of quantum confinement in the buckypaper/Parmax composites^39^. At the low temperature range (<50 K), the TEP linearly increase for both the composites and the buckypaper strip. But the TEP curves of the composites show a plateau between 100 K and 250 K, slightly decrease above 250 K. The S value of *60BPmx* drops to 22 μV/K at room temperature. The large positive S value of the buckypaper strip at T > 300 K might be attributed to the native oxygen doping in the tubes that shifts the Fermi level below the band crossing point^40^, while the oxygen dopants might lose during the high temperature processing of the composites. The ZT of our composites can be further improved by optimizing the buckypaper-to-Parmax ratio, the CNT length/diameter, bundle structures, doping level, and functionalization.

### Tensile Properties

**Figure 3:**
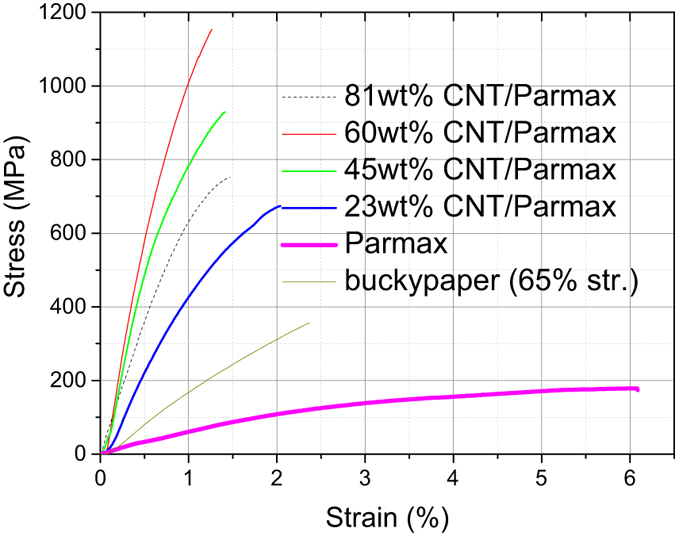
The typical uniaxial tensile stress–strain curves of the 65%-stretched buckypaper/Parmax composites with different nanotube concentration. The mechanical properties of the 65%-stretched buckypaper and the neat Parmax are also included for comparison.

The content effects of MWNT sheets on the tensile performances of the buckypaper/Parmax composites are shown in Fig. 3 and summarized in Table 1. The Young’s modulus and tensile strength for the neat Parmax are approximately 5.2 GPa and 178 MPa, respectively. As the framework and starting materials of the composites, the 65%-stretched buckypaper (*Str60BP*) strip demonstrates a 423 MPa tensile strength and a 21.6 GPa Young’s modulus, much higher than the pre-stretched buckypaper (*rndmBP*), but still much lower than individual CNT mainly because of the weak load transfer between CNTs in the buckypaper.

The incorporation of a proper amount of Parmax into the aligned buckypaper can dramatically improve the tensile properties. The elongation at the break (a direct indicator of a material’s toughness) decreases sharply with the increasing CNT content, due to an increased restriction of Parmax chain mobility under the presence of CNTs. This work shows that the tensile modulus, tensile strength, and elongation at break don’t have a monotonic trend with the buckypaper content. The tensile performance increases with the buckypaper content up to ~60 wt%. The composite *60BPmx* exhibits Young’s modulus and tensile strength of 150 GPa and 1145 MPa, respectively, outperforms structural metals, on par with thermoset composites, given its low mass density (~1.1 g/cm^3^, Table 1). Thanks to the high-loading aligned long tubes in the buckypaper/Parmax composites, the mechanical performance of our optimized composite is several times better than that of the recently reported CNT mat/pCBT thermoplastic composites^41^. Thus, these aligned buckypaper-reinforced Parmax composites can provide sufficient strength or stiffness for many applications, and have evolved to offer unprecedented strength-to-weight merits. Since the buckypaper/Parmax composites are relatively easy and inexpensive to process, they would be able to replace the widely used metal structural materials, which are heavy and subject to corrosion and fatigue.

For a neat buckypaper, the axial stress in the CNTs is built up by stress transfer between adjacent CNTs through shear and is thus proportional to CNT length. For given length of tubes, the maximal alignment of nanotubes in buckypaper is crucial for realizing a high degree of contact between the neighboring CNTs and load-transfer efficiency. Since the axial stress stems from the resistance to nanotube pull-out and the load transfer mainly works through the shear between the contacting CNTs, the stress transfer between adjacent tubes is proportional to the tube-tube interfacial contact area and shear strength. So the tube volume factor *f*_*v*_ in the previous widely used model cannot correctly reflect the contact area. Here we introduce an interpolating factor *f*, similar to the model proposed by Lielens and co-workers that interpolates between the upper and lower bounds^42^. In the most cases where individual CNTs are shorter than the gauge length (*η*_*l*_* = l*/*L < 1*) and don’t break under the tensile test, the Young’s modulus of a buckypaper can be expressed as:


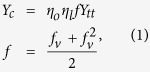


where *η*_*o*_ and *η*_*l*_ factors ranging from 0 to 1 correct the effects of the nanotube orientation and length, respectively. *Y*_*tt*_ is the modulus of CNT bundles. When the CNTs are well aligned and organized with long-range order^43^, the porosity of the buckypaper (1 − *f*_*v*_) would significantly reduce, the buckypaper can to large extent transfer many of the extraordinary properties from CNTs and offer opportunities for the promising real-world macroscopic structural applications.

Many composite studies focus on the low CNT-loading (<10 wt%) composites, in which the nanotubes can be relatively easily isolated and well dispersed in the polymer matrix, so that the nanotube aggregation effects can be neglected. In these cases of the low tube volume fraction ( *f*_*V*_ < 10%) composites, the Young’s modulus *Y*_*c*_ and strength *σ*_*c*_ of the composite are often assumed to be linearly scaled with the CNT volume fraction *f*_*V*_ as predicted by the rule of mixtures^44^:


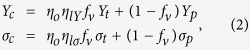


where *Y*_*T*_ and *Y*_*P*_ are the nanotube and polymer moduli respectively. Note that *η*_*lY*_ applies specifically to the composite modulus while a different length efficiency factor (*η*_*lσ*_) applies to the composite strength^45^. In the most cases the polymer moduli are very small and neglectable, so the composite moduli and strength can be simplified as *Y* = *η*_*lY*_*·η*_*o*_ *·f*_*v*_*·Y*_*T*_ and σ = *η*_*lσ*_*·η*_*o*_ *·f*_*v*_*·σ*_*T*,_ respectively. However, the above equation set 2 neglects the contribution of the interfacial interaction between tube and polymer. Generally this holds for the composites with a low CNT content.

For the high-CNT-content composites the tensile/shearing contribution from the interfacial interaction between tubes and polymer cannot be neglected. In the tensile test for the buckypaper/Parmax composites, the individual tube and polymer didn’t break in the process of tensile test. Thus the simple mixture model for the low filler composites doesn’t apply here^46^. The interfacial interactions of tube-polymer (σ_tp_), tube-tube (σ_tt_) and polymer-polymer (σ_pp_) are the major contributions to the tensile strength of the buckypaper/Parmax composites. Thus the modulus (Y_c_) and strength (σ_c_) of the hybrid composites can be approximated as:


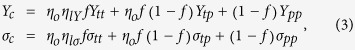


where the *f*(1-*f*) term in the equation set 3 takes into account the interaction between constitutes and the effect of the free boundary of the specimen^47^. The equation set 3 can hold for the composites with any CNT/Parmax volume ratio, *i.e*., the interpolating factor can range from 0 (pure Parmax) to 1 (pure buckypaper). In the case of pure buckypaper where no polymer presents *Y*_*tp*_ = *Y*_*pp*_ = 0, 1-*f* can be viewed as the fraction of the void space in buckypaper, just like in the equation 1. Additionally, the equation set 3 may also apply in other composite systems even with CNT aggregation.

The Young’s modules of the composites reaches its maximum when its first order derivative related to the interpolating factor *f* is zero, *i.e*.,


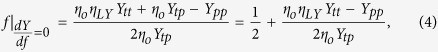


In our case the optimal CNT content is around 60 wt% for the Parmax matrix, the maximum tensile performance of the resulting composite is much higher than those of the neat buckypaper and Parmax. From the below fracture mechanism, the failure of neat buckypaper and Parmax are due to their weak interfacial interactions between tubes in neat buckypaper or polymer chains in neat Parmax. Incorporating CNTs into Parmax, the load transfer contributed from the interfacial interaction between the tubes and Parmax chain increases with the CNT loading, and this kind of contribution reaches its maximum at the CNT loading around 60 wt%. In the composite *60BPmx*, the interfacial interactions (*Y*_*tp*_ = 649 ± 60 GPa) between CNT and Parmax chains are much stronger than those of the neat buckypaper (*Y*_*tt*_ = 596 ± 60 GPa) and Parmax (*Y*_*pp*_ = 5.1 ± 0.5 GPa), based on the fitting results using the equation set 3 with the interpolating (0.57), tube orientation (0.86) and length factors (0.04) of *60BPmx*. Further increasing the CNT content in the composite would lead to fewer of the nanotubes having a chance to interact well with Parmax chains, so too high content of CNT in the composites does not mean high mechanical performance.

**Figure 4:**
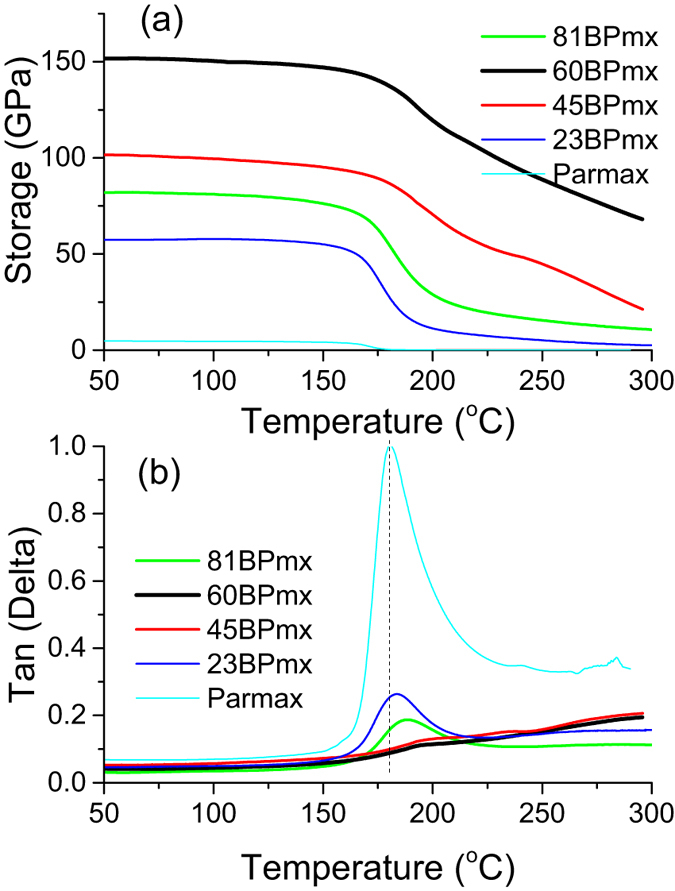
The storage modulus (**a**) and tangent(δ) (**b**) curves of the buckypaper/Parmax composites as a function of temperature.

In terms of mechanical performance, dynamic mechanical analysis (DMA) was carried out to confirm the measured stiffness and measure the glass transition temperature (*T*_*g*_) values of buckypaper/Parmax composites (Fig. 4). The composites exhibit higher storage modulus than the Parmax at all the test temperatures, indicating that the nanocomposites are more rigid than Parmax^48^. Additionally the storage modulus of the hybrid composite increases with increasing nanotube loading, suggesting that the CNTs enable the Parmax matrix to sustain its stiffness at a higher temperature range (near the *T*_*g*_), similar to the observation that the storage modulus of the HDPE increases with the CNT loading^49^. The storage moduli of the buckypaper/Parmax composites are well consistent with the Young’s modulus values in the tensile test. The *60BPmx* composite (60 wt% MWNT), for example, shows an increase in storage modulus of approximately 27 time (124.7 GPa) at room temperature compared to the neat Parmax (4.5 GPa). As suggested earlier^50^, the CNTs may be “wrapped” by polymer chains during melt-mixing under a high shear field at high temperature, forming a stable polymer-nanotube interface. The increased storage modulus is mainly attributed to the effect of the homogeneous dispersion of MWNTs in the Parmax matrix coupled with the significantly enhanced adhesion between the MWNTs and the matrix.

The polymer microstructure and crystallinity also affect the glass transition temperature of composites^51^. The glass transition temperature of polymer reflects the mobility of polymer chains. As shown from the peak temperature in the energy dissipation curves or tan(*δ*) curves, the *T*_*g*_ values of the composite *60BPmx* (196 °C) increases by 16 °C from the neat Parmax (180 °C). It indicates that the MWNTs hinder the polymer chain mobility^52^. The effective attachment of Parmax to nanotubes constrains the segmental motion of the polymer chains by the electrostatic attraction, accordingly increasing *T*_*g*_. The reduction of the tan(δ) areas of the buckypaper/Parmax composites with their storage modulus might suggest more molecular interactions between MWNTs and Parmax due to the spreading of the CNT bundles and large interface areas.

### Microstructure Analysis

#### The Fracture Morphology of the Composites

**Figure 5:**
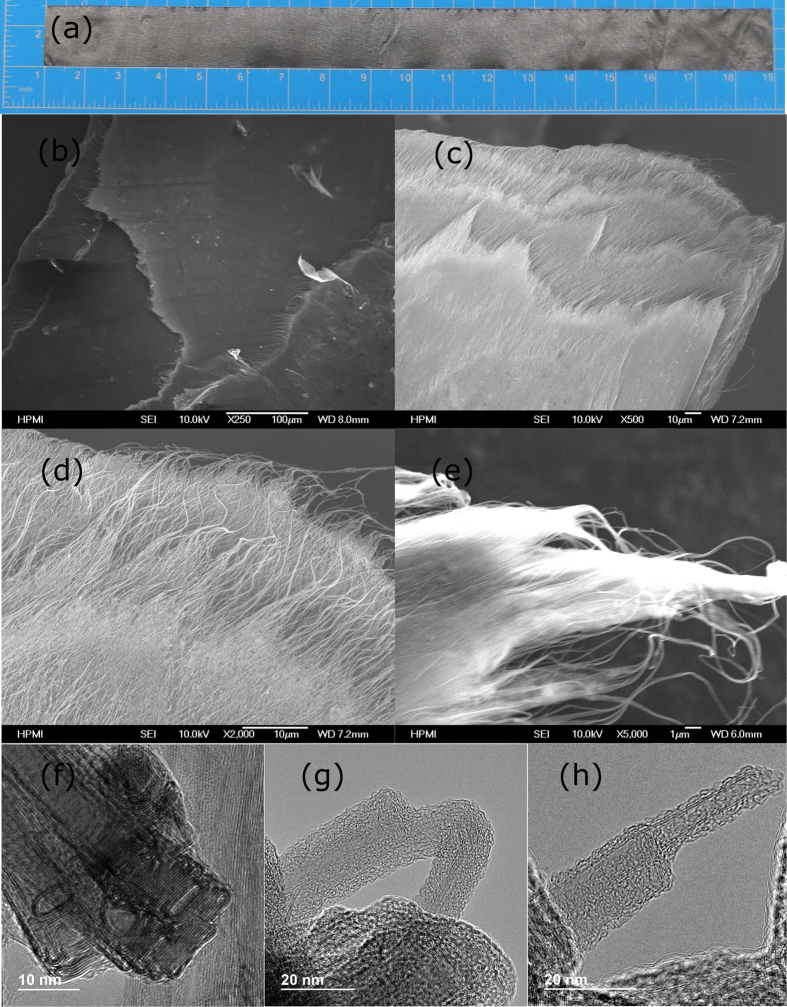
The optical photograph (**a**) of a buckypaper/Parmax composite strip cut from a 30 cm × 45 cm plate. (**b**–**e**) display the SEM morphology of the cross-sectional fracture surface of the 60BPmx composite after tensile testing at different magnification. The TEM image of the neat buckypaper (**a**) shows the flattened and stacked tubes, and that of the hybrid composite (**b**) clearly indicates the strong adhesion of polymer on the flattened tubes.

Using buckypaper as starting material enables to fabricate large composites for real applications (Fig. 5a). The microstructure of the buckypaper/Parmax composites was analyzed using high resolution SEM to understand the failure in the tensile test. The SEM images at different magnification (Fig. 5b and e) clearly reveal nanotube bundle telescoping, interlayer separation, and bundle pullout, resulting from relatively weak CNT/CNT and polymer/polymer interactions. The cross-sectional fracture SEM images of the *60BPmx* composites after tensile test show that the Parmax matrix homogeneously covers the aligned CNTs, suggesting good wetting and adhesions of the Parmax with the CNTs. Good dispersion and interfacial stress transferring are important factors for preparing reinforcing nanocomposites, benefiting a more uniform stress distribution and minimizes the presence of the stress concentration center^53^. The uniform dispersion and interfacial stress transferring play an important role in preparing reinforcing nanocomposites, leading to a more even stress distribution and minimizes the presence of the stress concentration center^54^. It further verifies our newly developed solution impregnation technique offer the better dispersion quality of Parmax on buckypaper than the previous reported stacking one^55^. The TEM image of the neat buckypaper (Fig. 5f) displays interesting structural features of unusual CNT crystal packing and their assemblages, including collapse, flattened packing, and preferred stacking. After the tensile test, CNT pullouts from bundles and the resin matrix, and Parmax coating remains staying on the tube wall (Fig. 5g,h), *consist with the above calculation results*. The large surface-to-surface contact areas between Parmax and flattened nanotubes, driven by π-stacking and CH-π interaction (see the FTIR section below), give rise to a high density packing of the “brick-and-mortar” structure in the nanocomposite, resembling a nacre material. On the fracture surfaces of the CNT-Parmax composites, however, no broken CNTs are observed, indicating that the load transfer from polymer to CNT is not sufficient to fracture individual tubes. Instead, the failure of the composite appears to arise from pullout of the nanotubes. The presence of a polymer layer on a nanotube after fiber pullout can indicate a strong filler-matrix interface. The actual interfacial energy for a given composite could be estimated by measuring the contact angles between nanotube and polymer. However, given the small diameter of the nanotubes and the lack of a robust testing platform at the nanoscale, it is difficult to reliably measure the critical interfacial parameters such as shear strength. Moreover, a comprehensive theory connecting nanoscale interfacial features to macroscopic properties is expected to be developed with the future breakthrough in the field of multi-scale modeling and quantitative nanomechanical characterization of CNT/Parmax interfaces.

#### Small and Wide Angle X-ray Scattering Microstructure Analysis

**Figure 6:**
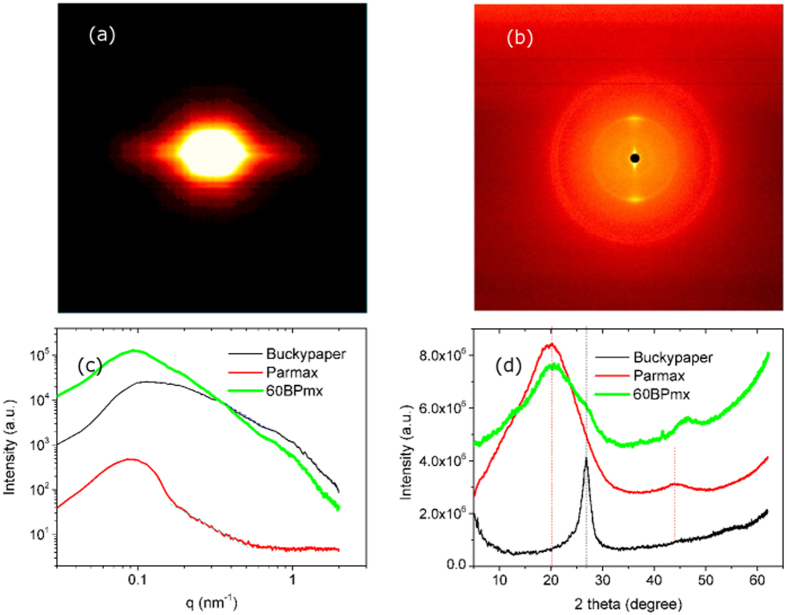
The (**a**) SAXS and (**b**) WAXS 2D patterns of the composite 60BPmx; The SAXS (**c**) and WAXS (**d**) comparison plots of the buckypaper, Parmax and 60BPmx composite.

Small (SAXS) and wide (WAXS) angle X-ray scattering techniques provide the Fourier transform of real space structural information and can be used to study the orientation and dispersion of CNT bundles, average diameters of individual CNTs and the deformation of CNT nanocomposites. As seen from the typical two dimensional (2-D) SAXS and WAXS patterns of the *60BPmx* (Fig. 6a,b), the distinct clouds (close packing peak) at small scattering angles localized near the horizontal axis and the narrow Bragg arcs from a strong diffraction of the (002) crystal planes of MWNTs at large scattering angle (26.7°) indicate high alignment of the longitudinal axes of the nanotubes in the composites with respect to the incident beam^56^. The arcs are characteristic of X-ray patterns from a uniaxial oriented specimen. The orientation degree of the respective scattering planes is directly reflected on the azimuthal width of the arcs. The relative intensity along the azimuthal, (*I*(ϕ)), at 2Θ is related to the orientation distribution function (ODF) of the scattering planes. Approximating the ODF as a Legendre polynomial series in cos ϕ, the Herman’s orientation parameter *S*_*d*_ (sometimes referred to as P_2_) is the second moment average of the ODF and its relation with alignment factor can be expressed as^57^:


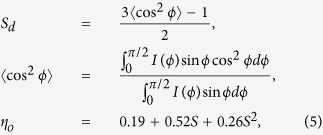


For uniaxial orientation, *S*_*d*_ values range from −0.5 to 1. The values of −0.5, 0 and 1 reflect perfect the alignment in the plane perpendicular to the uniaxial direction, random orientation, and alignment along the uniaxial direction, respectively^58^.

To characterize the long range alignment and anisotropy, the scattered X-ray counts are summed over the (002) diffraction peaks at the high angles of 20° < 2θ < 30°. Since the information obtained from X-ray diffraction includes contributions from both the nanotube and other constituents, *e.g*., voids, metal catalyst, amorphous carbon, etc. the background intensity must be removed before the Herman’s orientation function can be applied. The fitted full width at half maximum of *60BPmx* nanocomposite is 14°, and the Herman’s orientation factor described above is calculated to be 0.87. Similar to the pCB/buckypaper composites^59^, the alignment (Herman’s orientation factor) of carbon nanotubes in the composite changes with the different composition of MWNT and polymer, due to the CNTs/bundles sliding under hot-press caused by the molten Parmax. Figure 6c displays azimuthally integrated SAXS intensities versus scattering vector *q* extracted from the 2-D SAXS patterns. The intense small-angle signal can be assigned to both the form factor (*F(q*)) of the isolated nanotubes in the sample and the structure factor (*S(q*)) of the bundles with various size and shape. The feature in the range of *q* ≈ 1.0 nm^−1^ can be interpreted to correspond to the characteristic outer diameter of the MWNTs^60^. As seen in Fig. 6d, Parmax exhibits a wide peak centered at 19.93°^61^, corresponding 0.445 nm *d*-spacing. A strong diffraction of the (002) crystal planes of MWNTs shows at 26.7°, with a 0.336 nm calculated *d*-spacing^62^.

#### FTIR Analysis of Interfacial Interaction

**Figure 7:**
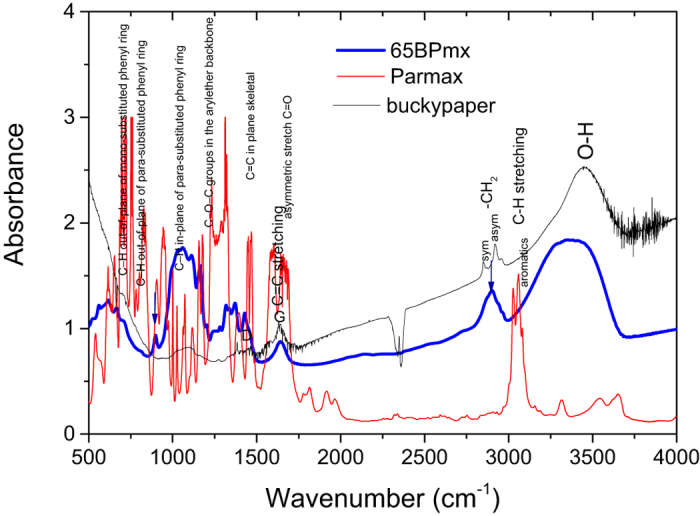
The FTIR spectra of Parmax, buckypaper and their composite (60BPmx). The notable change is the aromatic C-H stretching peaks around 3025 and 3050 cm^−1^ from the Parmax disappears in the composite, which might indicate the benzene rings of Parmax chain are well contact the wall of carbon nanotube (CH–π), and the red-shift of the vibration at 904.46 cm^−1^ in Parmax to 897.71 cm^−1^ in the composite was caused by the π–π stacking interaction in the Parmax-nanotube complex.

Three major modes of interaction between a CNT and a polymer matrix are chemical bonding, nano-mechanical interlocking, and weak interactions (van der Waals and electrostatic). A perfect *sp*^2^ hybridized carbon structure limits the formation of strong covalent bonds with a surrounding polymer matrix. Nano-mechanical interlocking could be difficult in nanotube composites due to the atomically smooth surface of aligned carbon nanotube. To further understand the interfacial interaction at molecular level, FTIR measurement in transmission mode was implemented for the buckypaper, Parmax and their composite. In Fig. 7, the = C–H stretch in aromatics is observed at 3100–3000 cm^−1^, which is at slightly higher frequency than that of the –C–H stretch in alkanes. Aromatic hydrocarbons show absorptions in the regions 1600–1585 cm^−1^ and 1500–1400 cm^−1^ due to carbon-carbon stretching vibrations in the aromatic ring. Bands in the region 1250–1000 cm^−1^ are due to C–H in-plane bending, although these bands are too weak to be observed in most aromatic compounds. Besides the C–H stretch above 3000 cm^−1^, two other regions of the infrared spectra of aromatics distinguish aromatics from organic compounds that do not have an aromatic ring: 2000–1665 cm^−1^ (weak bands known as “overtones”) and 900–675 cm^−1^ (out-of-plane or “oop” bands). The overtone bands in the region 2000–1665 cm^−1^ reflect the substitution pattern on the ring. The oop C–H bending bands in the region 900–675 cm^−1^ are also characteristic of the aromatic substitution pattern. The *MWNT* samples show a strong and broad peak around 3430 cm^−1^, which might correspond to the stretching mode of the O–H group introduced during the process^63^. The bands around 2950 and 2830 cm^−1^ are attributed to the asymmetric (*a*CH_2_) and symmetric (*s*CH_2_) stretching of C–H bond and the peak at 1630 cm^−1^ is due to the C=C stretching mode^64^,^65^. The FTIR valleys around 669 cm^−1^ and 2357 cm^−1^ should be assigned to CO_2_ which might be caused by the background subtraction.

After the buckypaper and Parmax formed a composite, the notable change in its FTIR spectrum is that the aromatic C-H stretching peaks around 3025 and 3059 cm^−1^ from the Parmax downshifts to 2901 cm^−1^ in the composite. It indicates that some Parmax chains should be absorbed to the CNT surface by forming weak hydrogen-bond-like CH–π interaction in nanocomposites^66^,^67^. Parmax plays as CH donor and sp^2^ sidewall of CNT plays as conjugated structure donor. The magnitude of CH–π interactions is determined by both the orbital distance and the orientation of Parmax chain and nanotubes^50^. The CH–π interaction is the key for Parmax coating the surface of CNT, and is also the incentive for the possible preordering chain conformation. Similarly the vibration at 904.46 cm^−1^ of Parmax redshifts to 897.71 cm^−1^ in the composite. The red-shift of this peak was caused by the stronger π–π stacking interaction in the Parmax-nanotube complex than in Parmax chain, and consequently the loosening of the C–H bond in the complex^68^.

**Figure 8:**
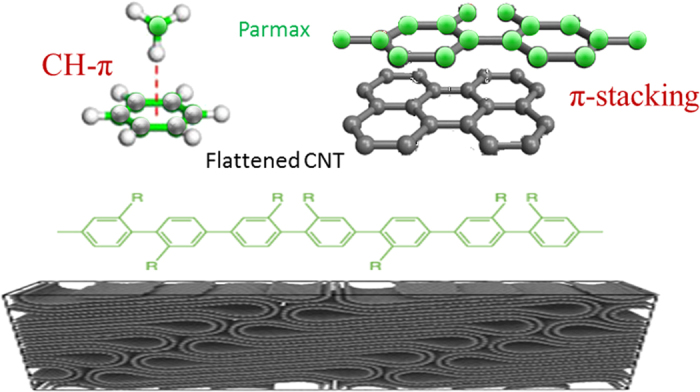
The schematic diagram of interfacial interaction between Parmax polymer chain and the flatterned CNT.

CNTs in the composites experience collapse, flattened high density packing (resembling a graphitic material), preferred stacking, folding under the high pressure compression during the hot-press fabrication process, which reduces the gaps between tubes and increases the surface contact area between tubes and polymer^15^,^69^. Parmax chains are highly aromatic, and this high degree of aromaticity attributes to the high chain stiffness^70^. Therefore, the aromatic ring structure of Parmax enables it to strongly interact with the nanotube walls through intermolecular overlap π-stacking. The schematic diagram of the interfacial interaction between Parmax polymer and the flattened CNT is displayed in Fig. 8. The complex hierarchical structures are built from layered/flattened CNT inorganic “bricks” interlinked through a small amount of organic “mortar” in between. Like the “brick-and-mortar” structure of nacre, such heterogeneous reinforcement architectures result in increased strengths and material properties exceeding those of the individual components alone. The *π*-stacking and CH-*π* interaction would enhance the good dispersion and interfacial stress transferring which are important factors for preparing reinforcing nanocomposites, leading to a more uniform stress distribution and minimizes the presence of the stress concentration center as well as better photon and charge transport at the interface between the two components.

## Conclusion

Ultimately, by mimicking the “brick-and-mortar” structure of nacre, we developed a facile method for the mass production of multifunctional thermoplastic composites by impregnating an aligned buckypaper framework with self-reinforcing polyphenylene, followed by a hot-press process. We also investigated the effects of buckypaper content on the mechanical performance, electrical and thermal properties of the composites. To determine the optimal filler/polymer ratio, by considering the contributions of the interfacial interactions, we developed a general empirical expression to describe the influence of the alignment, volume fraction, and nanotube length factors on the tensile performance of the composites. At optimal buckypaper content around 60 ± 5 wt%, the tensile strength and Young’s modulus of the buckypaper/Parmax composites reach 1145 MPa and 151 GPa, respectively, many times higher than the Parmax and buckypaper. The presence of *π*-*π* and CH-π interaction between Parmax and flattened CNT as confirmed by the FTIR analysis would enhance the good dispersion and interfacial stress transferring which are important factors for preparing reinforced nanocomposites. Polymer-tube interactions are more intense than those between nanotubes or polymer chains. The optimized composites are lightweight and outperform many structural materials. Several factors might synergistically contribute the performance: (1) the optimal composition ensures a large contact area between Parmax polymer and MWNTs, which accordingly improves the thermal power (Seebeck coefficients); (2) the strong interfacial adhesion due to π-stacking interaction between Parmax and flattened nanotube walls enhances the interfacial interaction between the Parmax and the CNTs; and (3) the highly aligned packing of the long nanotubes in the Parmax matrix improved the load transfer, which also significantly benefits the thermal and electric conductivities. Combined with a scalable fabrication procedure and its light mass density, the outstanding multifunctionalities of these bio-inspired composites make them very promising for numerous applications.

## Additional Information

**How to cite this article**: Li, Z. and Liang, Z. Optimization of Buckypaper-enhanced Multifunctional Thermoplastic Composites. *Sci. Rep.* **7**, 42423; doi: 10.1038/srep42423 (2017).

**Publisher's note:** Springer Nature remains neutral with regard to jurisdictional claims in published maps and institutional affiliations.

## Figures and Tables

**Figure 1:**
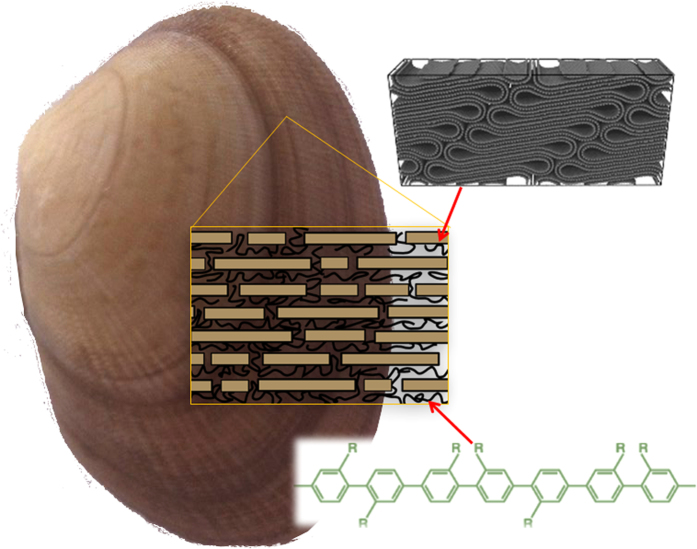
Schematic “bricks-and-mortar” structure diagram of nacre and the buckypaper/Parmax hybrid composite.

**Table 1:** Mechanical Properties of the 65%-stretched buckypaper/Parmax composites as a function of nanotube concentration.

Sample Name	Young’s Modulus (GPa)	Tensile Strength (MPa)	Elongation at Break (%)	Density (g/cm^3^)
rndmBP	1.7 ± 0.2	130 ± 11	23.9 ± 4.2	0.81 ± 0.05
Str60BP	21.6 ± 1.9	423 ± 37	2.37 ± 0.41	0.89 ± 0.05
81BPmx	87.1 ± 7.8	752 ± 64	1.47 ± 0.25	0.97 ± 0.05
60BPmx	150.7 ± 13.5	1145 ± 99	1.26 ± 0.22	1.05 ± 0.05
45BPmx	108.1 ± 9.7	929 ± 82	1.41 ± 0.24	1.08 ± 0.05
23BPmx	55.4 ± 4.9	673 ± 58	2.03 ± 0.35	1.12 ± 0.05
Parmax	5.2 ± 0.9	178 ± 15	6.08 ± 1.07	1.14 ± 0.05

The mechanical properties of the 65%-stretched buckypaper and the neat Parmax are also included for comparison.
